# Supplementary material for: Serial X-ray liquidography: multi-dimensional assay framework for exploring biomolecular structural dynamics with microgram quantities
Source: Nat Commun. 2024 Jul 26;15:6287. doi: 10.1038/s41467-024-50696-0 (PMC11282289; doi:10.1038/s41467-024-50696-0)
Supplement: Supplementary file 1 — Supplementary Information [file 41467_2024_50696_MOESM1_ESM.pdf]

Supplementary Information

Serial X-ray liquidography: Multi-dimensional assay framework for exploring biomolecular structural dynamics with microgram quantities

Seong Ok Kim[1,2], So Ri Yun[1,2], Hyosub Lee[1,2], Junbeom Jo[1,2], Doo-Sik Ahn[1,2], Doyeong Kim[1,2], Irina Kosheleva[3], Robert Henning[3], Jungmin Kim[1,2], Changin Kim[1,2], Seyoung You[1,2], Hanui Kim[1,2], Sang Jin Lee[1,2], Hyotcherl Ihee[1,2]*

[1]Center for Advanced Reactions Dynamics (CARD), Institute for Basic Science (IBS), Daejeon 34141, Republic of Korea.
[2]Department of Chemistry, Korea Advanced Institute of Science and Technology (KAIST), Daejeon 34141, Republic of Korea.
[3]Center for Advanced Radiation Sources, The University of Chicago; 9700 South Cass Avenue, Argonne, IL, 60439, USA.
*Corresponding author. Email: hyotcherl.ihee@kaist.ac.kr

This PDF file includes:

Supplementary Fig. 1 to 17
Supplementary Table 1 to 3
Supplementary Discussion
Supplementary References

Table of contents

Supplementary Fig. 1. Positive master layout.
Supplementary Fig. 2. Preparation of an SXL chip.
Supplementary Fig. 3. Sample loading scheme.
Supplementary Fig. 4. Photographs of the sample loading process.
Supplementary Fig. 5. Schematic representation of single and dual-probing schemes.
Supplementary Fig. 6. Static scattering curves of *At*UVR8 using the dual-probing scheme.
Supplementary Fig. 7. SXL data of *At*UVR8.
Supplementary Fig. 8. SXL data from various *At*UVR8 samples.
Supplementary Fig. 9: Comparison of the DS at 316 ms from SXL with the reference from the static SAXS curves from wild type and R146A/R286A mutant of *At*UVR8.
Supplementary Fig. 10: SXL data of *At*UVR8 as a function of time.
Supplementary Fig. 11. Comparison of three SADSs.
Supplementary Fig. 12. Comparison of SXL data of *At*UVR8 from multiple data collections using two SXL chips.
Supplementary Fig. 13. SXL data from *As*LOV2 at various sample environments.
Supplementary Fig. 14. Comparison of the SADSs between the second and third species in the photoreaction of *As*LOV2.
Supplementary Fig. 15. Photograph of a capillary containing the *As*LOV2 sample after a conventional TRXL measurement.
Supplementary Fig. 16. SXL data from lysozyme.
Supplementary Fig. 17. SAXS profiles and DSs of lysozyme under various pH conditions.
Supplementary Table 1. Summary of time constants of *As*LOV2 at various sample environments.
Supplementary Table 2. Sample preparation for the assay of the reaction dynamics under diverse experimental conditions.
Supplementary Table 3. List of plasmids and their corresponding protein sequences used in this study.
Supplementary Discussion
Supplementary References

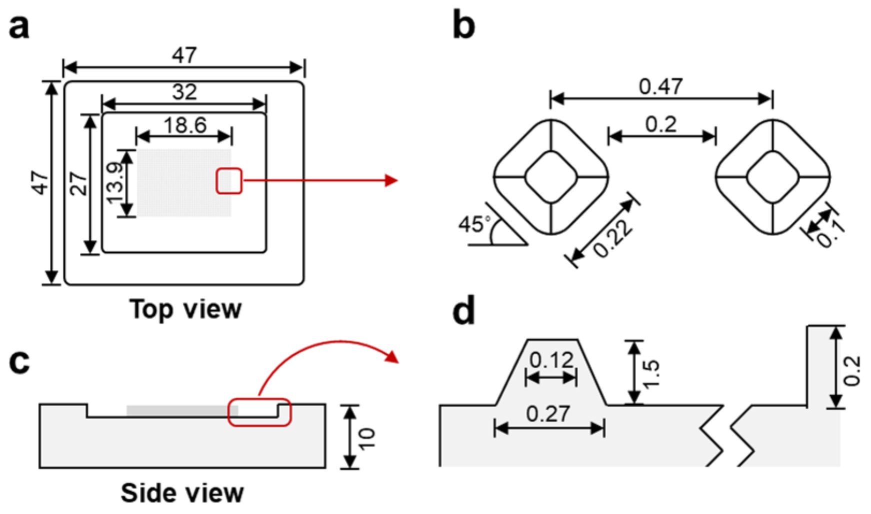

Supplementary Fig. 1. Positive master layout. The positive master was fabricated through a micro-milling process using ceramic-filled PEEK (CMF) material (**a**) Detailed dimensions (in mm) of the positive master from the top view. (**b**) Detailed dimensions reverse mold of the microchambers from the top view. (**c**) Detailed dimensions (in mm) of the positive master from the side view. (**d**) Enlarged details of the positive master from the side view. It should be noted that the outer architecture of the positive master has a height of 0.2 mm, which is 0.05 mm higher than the internal reverse mold for the microchambers, creating an asymmetric layout with the backside closing at a thickness of 0.05 mm.

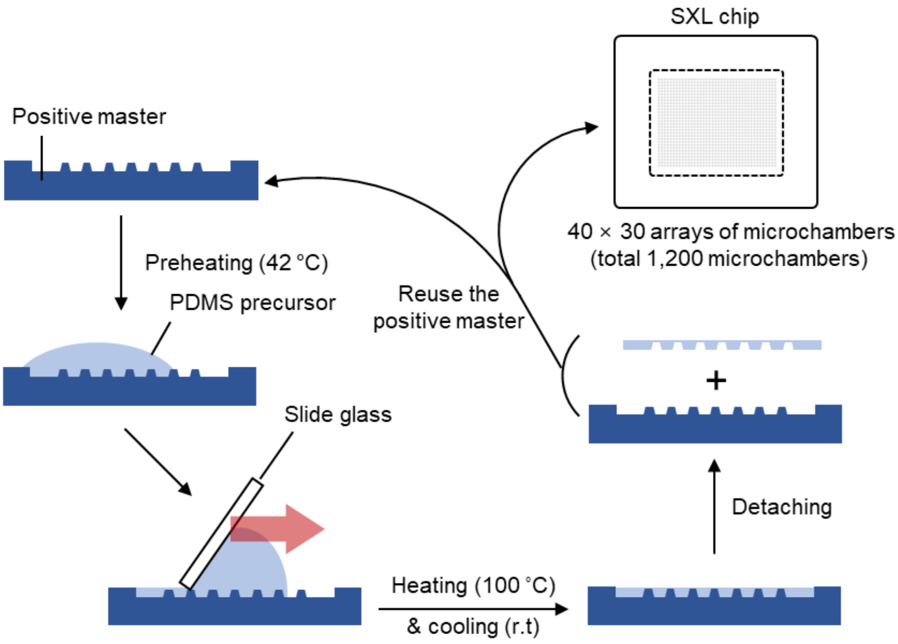

Supplementary Fig. 2. Preparation of an SXL chip. (i) The positive master and degassed PDMS precursor are preheated at 42 °C to reduce the viscosity of the precursor. The degassed PDMS precursor is poured onto the positive master. (ii) The excess amount of PDMS is removed by a slide glass. (iii) The PDMS precursor and the positive master are baked at 100 ˚C for 1 hour and cured at room temperature. (iv) The fabricated serial scattering chip is detached from the positive master.

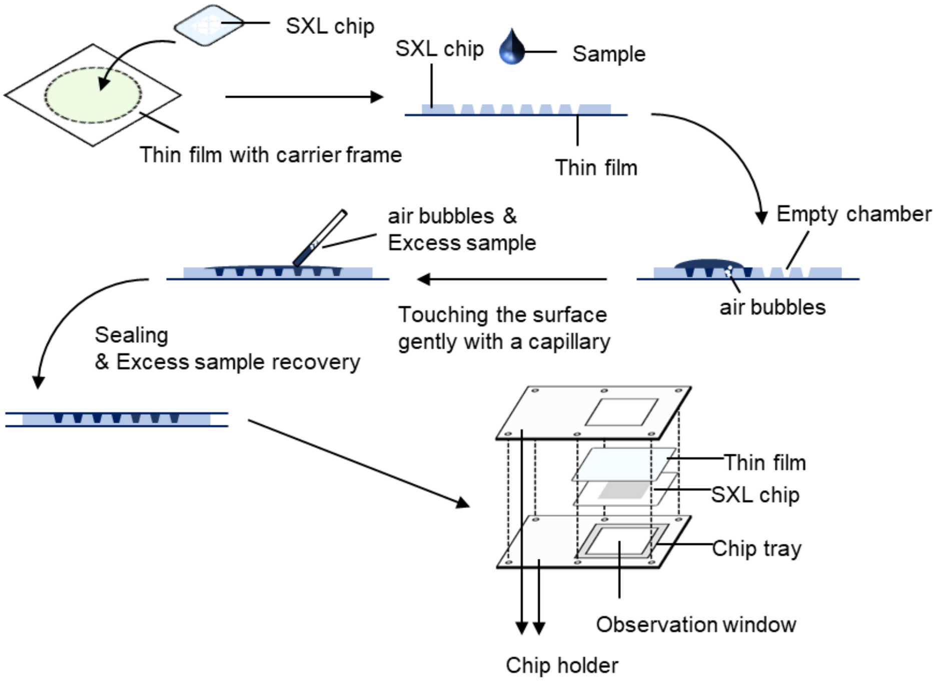

Supplementary Fig. 3. Sample loading scheme. (i) An SXL chip is placed on a transparent film. The solution sample is pipetted and spread to cover the microchambers. The thin film with the carrier frame enhances the structural integrity of microchambers and flattens the SXL chip. (ii) The surface of the microchambers containing air bubbles is gently touched with a round-shaped capillary to complete the sample loading process. (iii) The air bubbles are removed by the capillary effect. The excess sample solution is also recovered in this step. (iv) A new transparent film is carefully covered to seal the chip. (v) The sealed sample chip is assembled with an aluminum sample holder and mounted on the translate stage for data collection.

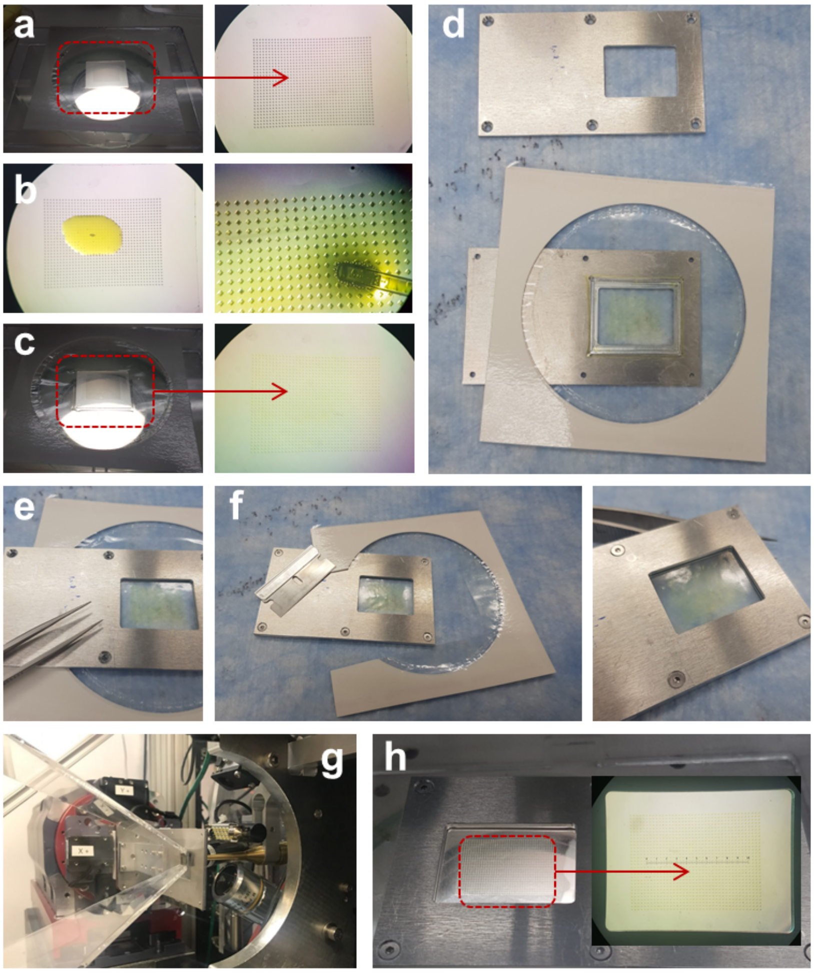

Supplementary Fig. 4. Photographs of the sample loading process. (**a**) A microscope image of the SXL chip. The SXL chip is placed on a transparent film which serves as a carrier frame, allowing for easy sample loading, flattening and enhancing the structural integrity of the chip. (**b**) The solution sample is pipetted and spread to cover the surface of the chip. Air bubbles are removed by gently touching the round-shaped capillary to complete the process. The air bubbles are removed by the capillary effect and the excess solution is recovered in this step. (**c**) (Left panel) A new transparent film is carefully covered, ensuring bubble-free loading. (Right panel) A microscope image of the sample loaded chip. (**d**) The sealed chip is placed on the chip tray in a sample holder. (**e**) Screws are tightened to assemble the sample holder. (**f**) The trace film and carrier frame are removed. (**g**) The sealed chip is mounted on the translation stage for data collection. (**h**) The photograph of the chip after data collection. (Enlarged section on the right) A microscope image of the SXL chip. It should be noted that the chip remains free from bubble formation or sample leakage.

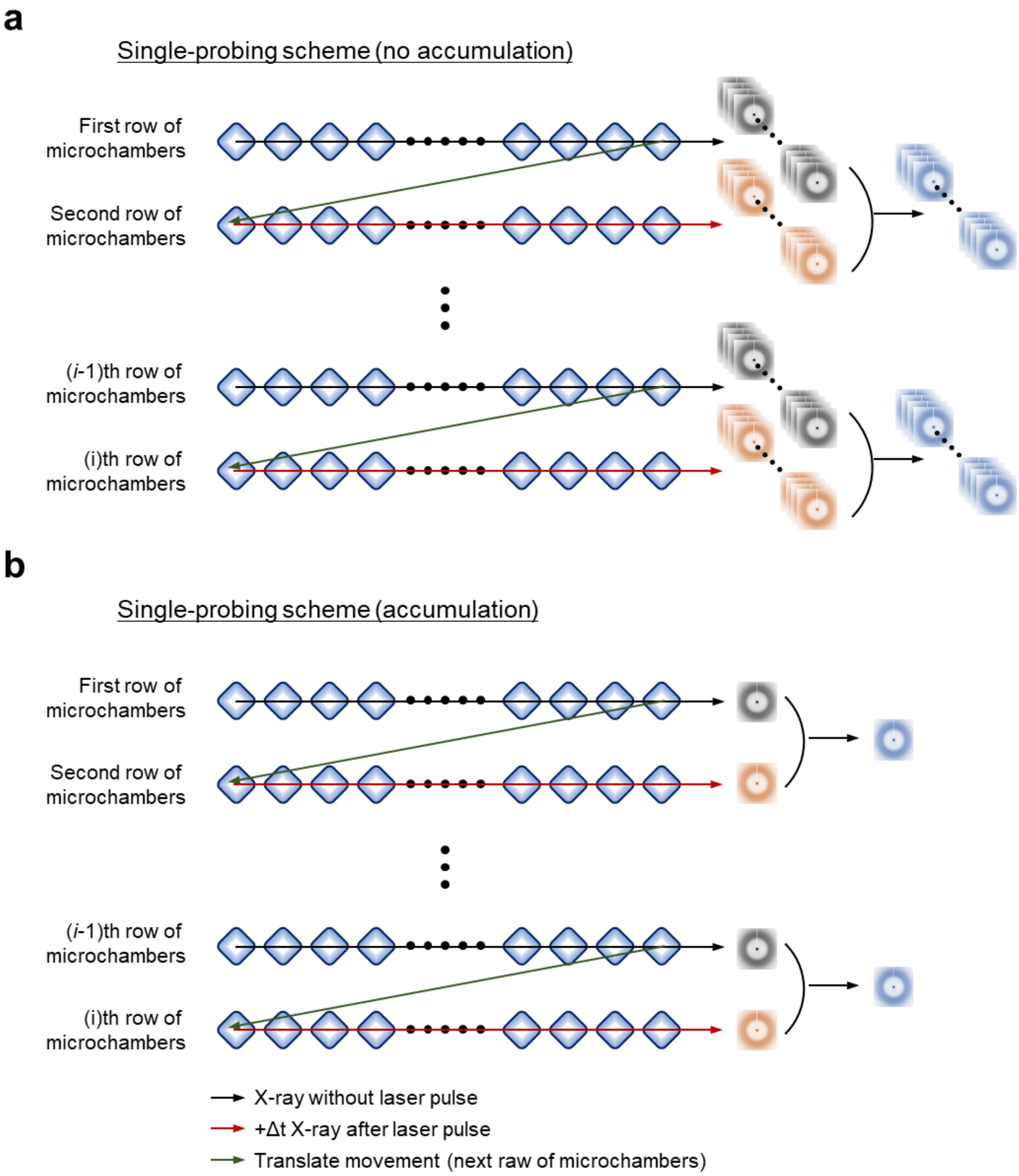
a
Single-probing scheme (no accumulation)
First row of microchambers
Second row of microchambers
(i-1)th row of microchambers
(i)th row of microchambers
b
Single-probing scheme (accumulation)
First row of microchambers
Second row of microchambers
(i-1)th row of microchambers
(i)th row of microchambers
X-ray without laser pulse
+Δt X-ray after laser pulse
Translate movement (next raw of microchambers)

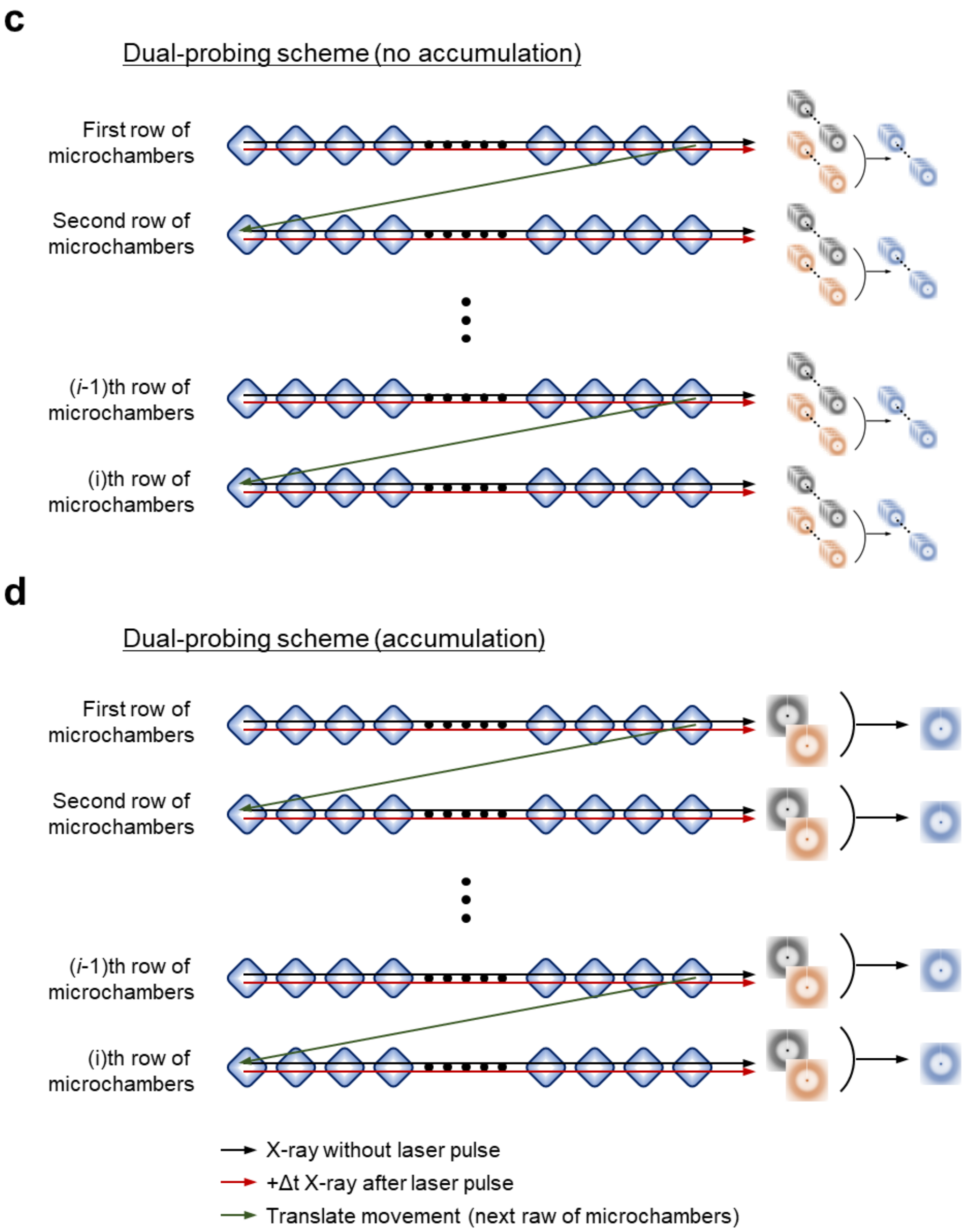

Supplementary Fig. 5. Schematic representation of single- and dual-probing schemes. Single-probing scheme without signal accumulation (**a**) and with signal accumulation (**b**). Dual-probing scheme without signal accumulation (**c**) and with signal accumulation (**d**). Microchambers are depicted as blue-colored rotated squares. The black arrows indicate the positional trajectories of data collection without a laser pulse, and the resulting X-ray scattering image is depicted in black color. The red arrows indicate the positional trajectories of data collection using both a laser and X-ray pulses, and the resulting X-ray image is depicted in red color. Subsequently, DSs are obtained by subtracting the scattering curves without and with laser pulses. In reality, the absolute positions of the laser and X-ray pulses remain constant whereas the positions of the microchambers are moved. In the single-probing

scheme, each fresh microchamber is used only once either without or with a laser pulse. In contrast, in the dual-probing scheme, each fresh microchamber is used twice; one without a laser pulse and the other with a laser pulse. In this study, we employed the single-probing scheme without signal accumulation (a) to investigate the intactness of individual microchambers. Additionally, we utilized the dual-probing scheme with signal accumulation (d) for both the pilot experiments and the main data collection.

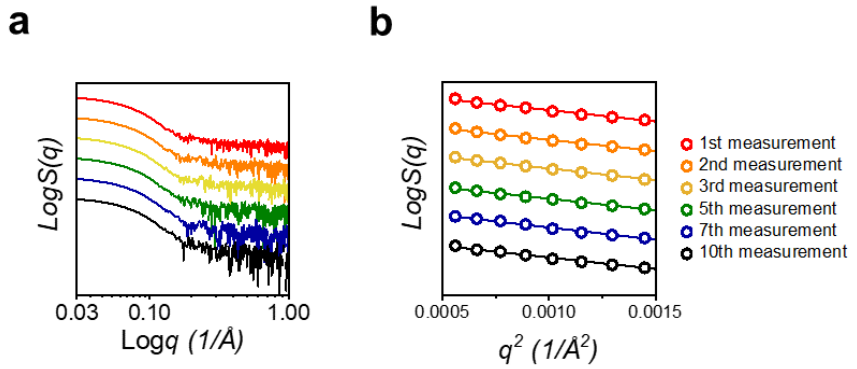

Supplementary Fig. 6. Static scattering curves of *At*UVR8 using the dual-probing scheme. Scattering curves (**a**) and Guinier plots (**b**) of *At*UVR8. The curves are vertically displaced for better visualization. The scattering curves do not show any noticeable abnormality, and the Guinier plots show a linearity as the number of X-ray exposures increases.

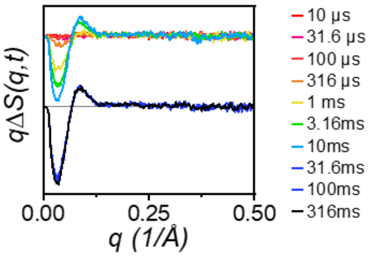

Supplementary Fig. 7. SXL data of *At*UVR8. SXL data of *At*UVR8 at the propagation (top) and saturation (bottom) stages. The difference signals propagate until the timescale of tens of milliseconds and become saturated after 31.6 ms.

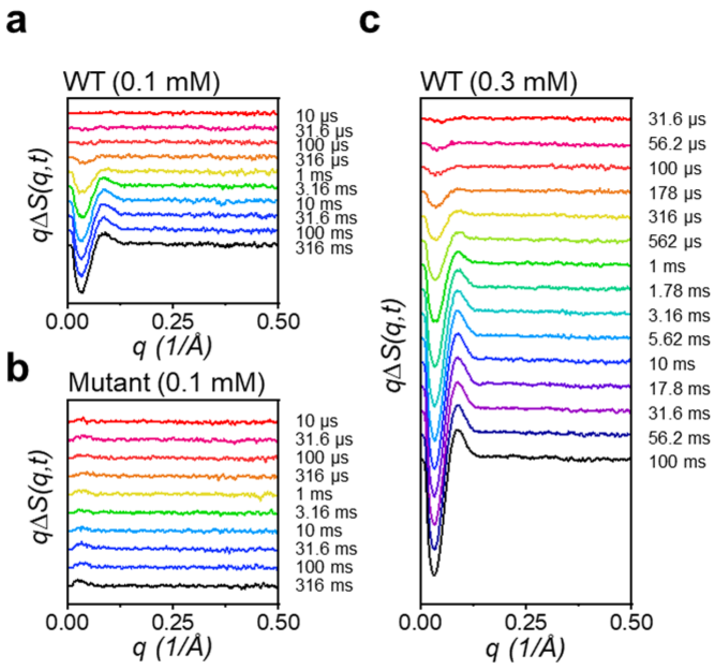

Supplementary Fig. 8. SXL data from various *At*UVR8 samples. (**a**) SXL data from the wild type of *At*UVR8 at 0.1 mM of protein concentration. (**b**) SXL data from the monomeric mutant of *At*UVR8 at 0.1 mM of protein concentration. It is noticeable that the intensities of the DSs from the mutant sample are weaker than those of the wild type. (**c**) SXL data from the wild type of *At*UVR8 at 0.3 mM of protein concentration. To improve the SNR, we averaged the SXL signals using two chips.

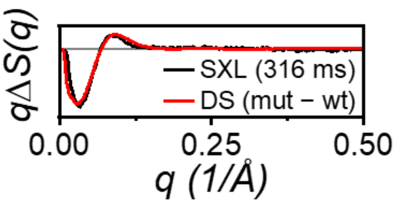

Supplementary Fig. 9. Comparison of the DS at 316 ms from SXL (black) with the reference from the static SAXS curves from wild type and R146A/R286A mutant of *At*UVR8 (red). It is notable that the features of DSs are comparable while the concentrations of the samples for SXL and static SAXS are different.

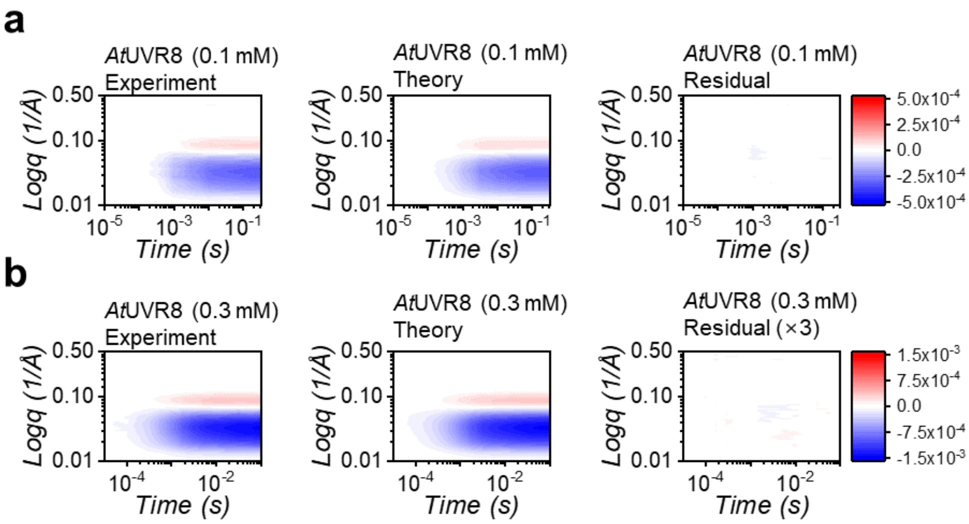

Supplementary Fig. 10. SXL data of *At*UVR8 as a function of time. (**a**) Experimental SXL data (Left panel) from the wild type of *At*UVR8 at 0.1 mM of protein concentration. Theoretical (Middle panel) and residual (Right panel) with respect to the experimental data are shown. (**b**) The counterpart of (a) with a different protein concentration (0.3 mM).

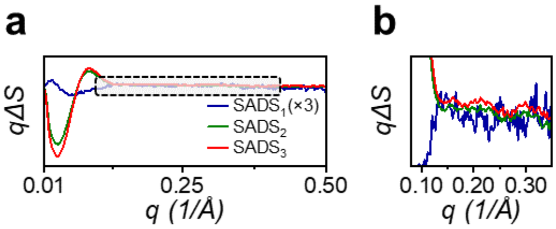

Supplementary Fig. 11. Comparison of three SADSs. (**a**) To compare the features of each intermediate, three SADSs are overlaid. (**b**) Enlargement of (a) in the q range of 0.1 to 0.35 Å^{-1}. The $SADS_3$ shares similar features with $SADS_2$, but it showed different intensity at the low q region.

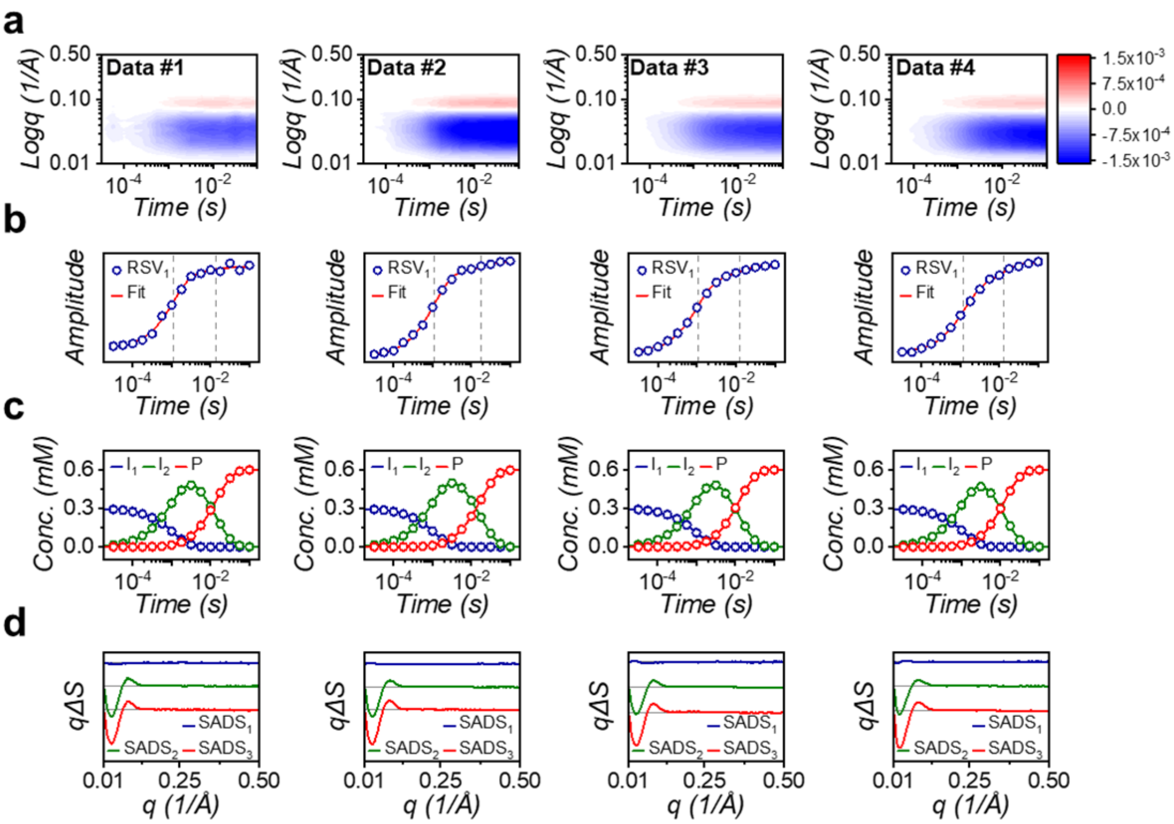

Supplementary Fig. 12. Comparison of SXL data of *At*UVR8 from multiple data collections using two SXL chips. (a) SXL data of *At*UVR8. Four sets of data were collected from two chips, with one collection cycle of time delays obtained from half of one chip. (**b**) Fits of the first RSV to obtain time constants of *At*UVR8 from each data collection cycle. (**c**) Obtained population changes of three intermediates in the photoreaction of *At*UVR8. (**d**) SADSs for the three intermediates. All obtained kinetic and structural information from each data collection is identical, indicating the stability and consistency of the SXL experiments. Naturally, the SNR of the dataset can be easily enhanced by collecting multiple sets of data with the same time delays from multiple SXL chips.

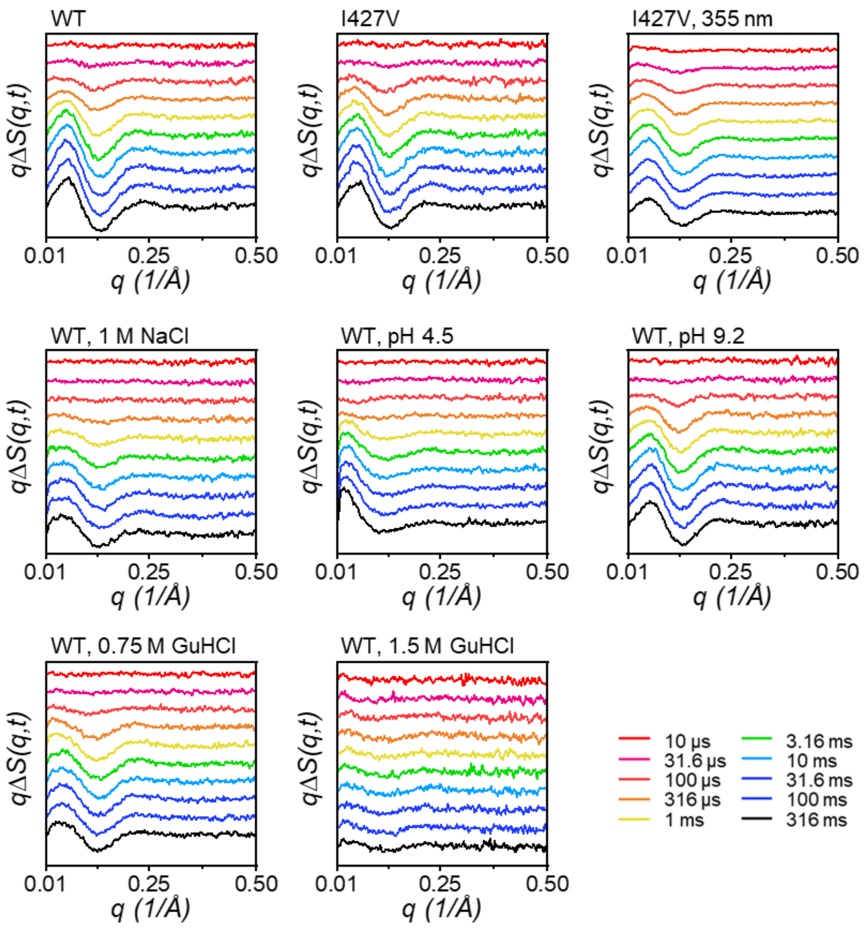

Supplementary Fig. 13. SXL data from *As*LOV2 at various sample environments. These data were used to show the 2D contour map in Figure 5. (Bottom right panel) Each time delay is colored as indicated.

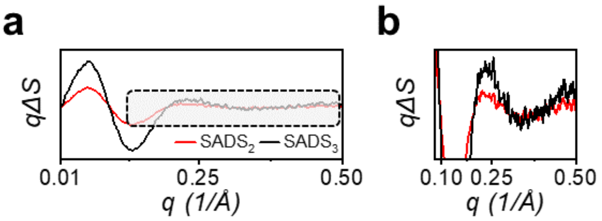

Supplementary Fig. 14. Comparison of the SADSs between the second and third species in the photoreaction of *As*LOV2. (**a**) The SADSs in the q range of 0.01 to 0.5 Å^{-1}. (**b**) Enlargement of (a) in the q range of 0.08 to 0.5 Å^{-1}. Although the two curves share a similar shape, the intensity of the curves in the low q region and the oscillation features in the high q region are slightly different, indicating an additional structural change occurs after forming the second species.

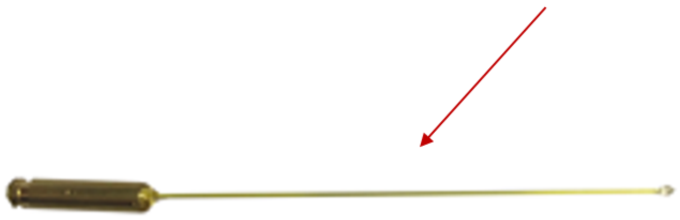

Supplementary Fig. 15. Photograph of a capillary containing the *As*LOV2 sample after a conventional TRXL measurement. The color of the sample irradiated by laser and X-ray pulses in the middle of the capillary became dark green due to sample damage. It is noteworthy that the color of the sample on the right side of the capillary, where the measurement was not carried out, remains the original fluorescent yellow.

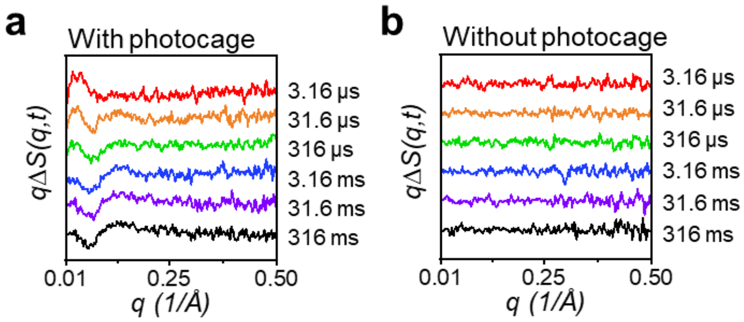

Supplementary Fig. 16. SXL data from lysozyme. SXL of lysozyme with (**a**) and without (**b**) NPE-caged proton. These data were used to show the 2D contour map in Figure 6.

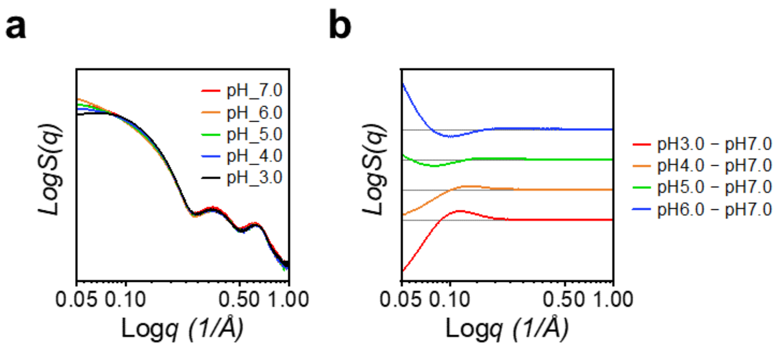

Supplementary Fig. 17. SAXS profiles and DSs of lysozyme under various pH conditions. (**a**) SAXS profile of lysozyme. (**b**) DSs between the various pH conditions and the neutral pH condition.

Supplementary Table 1. Summary of time constants of *As*LOV2 at various sample environments measured via SXL.

Samples	Conditions	Wavelength	t_1	t_2	t_3*
Wild type	Neutral pH, isotonic condition	450 nm	730 ± 60 μs	8.6 ± 2.8 ms	55 s
I427V mutant	Neutral pH, isotonic condition	450 nm	180 ± 20 μs	4.2 ± 0.7 ms	4 s
I427V mutant	Neutral pH, isotonic condition	355 nm	70 ± 20 μs	1.3 ± 0.3 ms	4 s
Wild type	Neutral pH, 1M NaCl	450 nm	650 ± 100 μs	10.4 ± 1.8 ms	-
Wild type	pH 9.2, isotonic condition	450 nm	410 ± 40 μs	6.3 ± 1.7 ms	55 s
Wild type	pH 4.5, isotonic condition	450 nm	1.2 ± 0.3	85.2 ± 70 ms**	55 s
Wild type	0.75 M GuHCl	450 nm	330 ± 20 μs	12.7 ± 7.4 ms	55 s
Wild type	1.5 M GuHCl	450 nm	110 ± 60 μs	-	-

*t_3 (recovery time) was fixed.

**It has been observed that the structural and kinetic aspects of *As*LOV2 under low pH conditions significantly differ from those in other conditions. The sample after the SXL experiment exhibits turbid-colored aggregates, suggesting instability of *As*LOV2 in acidic conditions. Consequently, the time constant of I_2 in acidic conditions exhibits a large error range.

Supplementary Table 2. Sample preparation for the SXL assay of the reaction dynamics under diverse experimental conditions

Sample name	Protein stock solution		Additive stock solution		Buffer volume (μL)	Final concentration of additive
	Type (Concentration)	Volume (μL)	Type (Concentration)	Volume (μL)		
Wild type	Wild type (2 mM)	30	None	0	10*	0
I427V	I427V mutant (2 mM)	30	None	0	10*	0
High Salt	Wild type (2 mM)	30	NaCl (5 M)	8	2*	1 M NaCl
High pH	Wild type (2 mM)	30	Tris, pH 9.2 (1 M)	10	0	200 mM Tris, pH 9.2
Low pH	Wild type (2 mM)	30	Sodium acetate, pH 4.5 (1M)	10	0	200 mM Sodium acetate, pH 4.5
0.75 M Guanidine-HCl	Wild type (2 mM)	30	Guanidine-HCl (6 M)	5	5*	0.75 M Guanidine-HCl
1.5 M Guanidine-HCl	Wild type (2 mM)	30	Guanidine-HCl (6 M)	10	0	1.5 M Guanidine-HCl
Photocaged reaction	Lysozyme (100 mg/ml)	30	Photocaged proton (50 mM)	8	2**	10 mM photocaged proton

*Buffer content: 5 mM Tris, pH 7.0, 150 mM NaCl

**Deionized water, pH 7.0

Supplementary Table 3. List of plasmids and their corresponding protein sequences used in this study

Name	Species<br>Protein name	Coding region	Protein sequence
2B-T-AsLOV2	*Avena Sativa*<br>Phototropin 1	404-546	LATTLERIEKNFVITDPRLPDNPIIFASDSFLQLT<br>EYSREEILGRNCRFLQGPETDRATVRKIRDAID<br>NQTEVTVQLINYTKSGKKFWNLFHLQPMRDQ<br>KGDVQYFIGVQLDGTEHVRDAAEREGVMLIK<br>KTAENIDEAAKEL
2B-T-AsLOV2 (I427V)	*Avena Sativa*<br>Phototropin 1	404-546	LATTLERIEKNFVITDPRLPDNPVIFASDSFLQL<br>TEYSREEILGRNCRFLQGPETDRATVRKIRDAI<br>DNQTEVTVQLINYTKSGKKFWNLFHLQPMRD<br>QKGDVQYFIGVQLDGTEHVRDAAEREGVMLI<br>KKTAENIDEAAKEL
2M-T-*At*UVR8-His	*Arabidopsis thaliana*<br>UVR8	12-381	APPRKVLIISAGASHSVALLSGDIVCSWGRGED<br>GQLGHGDAEDRPSPTQLSALDGHQIVSVTCGA<br>DHTVAYSQSGMEVYSWGWGDFGRLGHGNSS<br>DLFTPLPIKALHGIRIKQIACGDSHCLAVTMEG<br>EVQSWGRNQNGQLGLGDTEDSLVPQKIQAFE<br>GIRIKMVAAGAEHTAAVTEDGDLYGWGWGR<br>YGNLGLGDRTDRLVPERVTSTGGEKMSMVAC<br>GWRHTISVSYSGALYTYGWSKYGQLGHGDLE<br>DHLIPHKLEALSNSFISQISGGWRHTMALTSDG<br>KLYGWGWNKFGQVGVGNNLDQCSPVQVRFP<br>DDQKVVQVSCGWRHTLAVTERNNVFAWGRG<br>TNGQLGIGESVDRNFPKIIEALSVDG
*At*UVR8-R146A/R286A	*Arabidopsis thaliana*<br>UVR8	12-381	APPRKVLIISAGASHSVALLSGDIVCSWGRGED<br>GQLGHGDAEDRPSPTQLSALDGHQIVSVTCGA<br>DHTVAYSQSGMEVYSWGWGDFGRLGHGNSS<br>DLFTPLPIKALHGIRIKQIACGDSHCLAVTMEG<br>EVQSWGANQNGQLGLGDTEDSLVPQKIQAFE<br>GIRIKMVAAGAEHTAAVTEDGDLYGWGWGR<br>YGNLGLGDRTDRLVPERVTSTGGEKMSMVAC<br>GWRHTISVSYSGALYTYGWSKYGQLGHGDLE<br>DHLIPHKLEALSNSFISQISGGWAHTMALTSDG<br>KLYGWGWNKFGQVGVGNNLDQCSPVQVRFP<br>DDQKVVQVSCGWRHTLAVTERNNVFAWGRG<br>TNGQLGIGESVDRNFPKIIEALSVDG

Supplementary Discussion

Required sample quantities for irreversible reactions in conventional TRXL

While specifying a required sample quantity for irreversible reactions can be challenging, large sample consumption is a well-recognized obstacle for studying such reactions with time-resolved methods. This is exemplified by a previous TR-SAXS study, where the molecular association of the nucleotide-binding domain of MsbA was investigated using the photocaged ATP molecule[1]. In this study, the inherent nature of photolysis necessitated discarding the sample after each measurement. Based on the detailed description of sample usage provided in their Supporting Information, they utilized at least 45 milligrams of sample to collect TR-SAXS data at 13 time delays ranging from 50 ms to 1.4 s (3.5 mg per time delay). In comparison, for the irreversible photoreaction of *At*UVR8, SXL requires only 115 µg of sample to collect data at 15 time delays (7.7 µg per time delay). This estimation may underestimate the true sample consumption, as it only considers the sample used for the measurement and excludes additional waste generated by their flow-cell system, as well as the optimization of experimental conditions for data collection. While estimated sample usage may not accurately reflect the requirements of a general irreversible reaction, the substantial sample consumption observed in this study is a compelling illustration of the potential bottleneck this issue can pose, especially for studying diverse biological reactions, particularly irreversible ones. This is because biomolecule samples are often limited and difficult to prepare. As we have established throughout the text, one of the key advantages of SXL is its ability to utilize minimal sample quantities while still effectively exploring a wide range of biological reactions.

Drawbacks of conventional and potential alternative approaches to studying irreversible reactions

Existing methods such as closed capillary and flow-cell systems along with the droplet-on-demand (DOD) and liquid-jet approaches, were considered as potential alternatives to address the issue of high sample consumption. While these ideas may seem promising in terms of saving samples at the point of a single measurement, they are not free from systematic sample wastage and practical challenges, as described below.

The closed capillary system, although it allows for a small final volume, necessitates additional sample wastage during the filling process and faces difficulties in utilizing the entire sample content. In our work, we used a Hamilton syringe (approximately 50 µL of sample) to load the samples into capillaries with a diameter of 0.6 mm. Filling the capillary without air bubbles required approximately more than 30 µL of sample, and it was challenging to recover the sample remaining inside the needle and syringe. Moreover, utilizing the entire sample content within the capillary to obtain the TR signal is practically challenging due to the imperfectness of the capillary such as variations in curvature, roughness and diameter along the capillary. Due to surface roughness and inhomogeneous diameter distribution along the capillary, only 20 mm of the capillary region could be effectively utilized. In addition, the merging of TR data obtained from individual capillaries became complex due to the variations among capillaries, even when we successfully captured the TR signal from the entire sample content. For example, we encountered difficulties in obtaining an identical TRXL signal after realigning the capillary, showcasing the limitations of applying the conventional TRXL with closed capillary systems to irreversible reactions.

When considering the flow-cell system, it becomes evident that additional sample volume is required to fill up the pump and tubing parts. It should be noted that more than 80 µL of the sample solution is required to fill the connecting tube with a diameter of 1 mm and a length of 10 cm, and this volume is still insufficient to cover the whole flow-cell system. Maintaining a static flow without

pulsing effects and eliminating vibrational jitter from the movement of mechanical gears in the pump and rapid flow of sample in a small diameter of capillary, also proved to be challenging tasks. Moreover, conducting pilot experiments to determine the optimal flow rates and sample concentrations leads to sample wastage. Another severe limitation is the inability to cover the entire time window of reaction progress in a single batch due to the timing mismatch between the pump and probe pulses. Consequently, extra sample wastage is unavoidable to screen new experimental parameters within a specific range of time delays to encompass the entire reaction time window. Furthermore, delivering a viscous liquid sample poses difficulties and requires additional dilution, which subsequently lowers the SNR and data collection efficiency. We encountered similar challenges when attempting to collect TRXL data of *At*UVR8 using the flow-cell system. Several milligrams of *At*UVR8 sample were wasted in setting up the flow-cell system to fill the tubing and syringe pump and screen the optimal flow rate for delivering the sample solution to the capillary. However, we were unable to maintain a steady flow due to the high viscosity of the concentrated sample. Even when using a lower concentration of the sample, approximately 0.4 mL of the sample was consumed for the small-angle X-ray scattering (SAXS) experiment, significantly higher than the sample quantity required for the SXL experiment. Taking into account the additional wastage for sample delivery, the flow-cell system requires more than a hundred times the sample quantity of the SXL approach, making it unsuitable for studying irreversible reactions.

Liquid-jet systems focused by an outer stream of inert gas, similar to flow-cell systems, have a high potential to save unwanted sample waste since these setups have been well established over a decade and often used to explore the reaction dynamics in the ultrafast timescale[2-6]. However, these approaches share limitations that hinder their application to diverse biological reactions. While the high speed of the jet (i.e., repetition rate: 120 Hz, about 2.1 m/s[6]) is effective for studying the reaction dynamics of ultrafast reactions, a liquid-jet system requires a significant sample quantity per time delay. In contrast, SXL has the potential to significantly enhance efficiency by reducing the reaction chamber size to match the X-ray pulse. As discussed previously, this reduction could proportionally increase efficiency by the cube of the size reduction. This highlights the potential of SXL for future optimization while demonstrating its comparable efficiency in its current layout. The situation becomes even more evident when studying slower reactions in the milliseconds time range. At slower timescales, a liquid-jet system requires a significantly reduced repetition rate and thus necessitates the consumption of a tremendously larger sample quantity. This limitation arises due to the inherent constraints on slowing down the microjet's sample delivery speed. For instance, acquiring data at a 100 ms time delay using the liquid-jet system used at an XFEL beamline—operated at a 120 Hz repetition rate to elucidate ultrafast reaction dynamics[6]—would reduce the theoretical maximum repetition rate to 10 Hz, effectively decreasing the sample utilization efficiency to ~8% (= 10/120). In contrast, our SXL system readily adapts to such changes in experimental conditions without sacrificing sample utilization efficiency. This highlights the significant sample consumption required by the liquid-jet system at slower timescales.

Although the DOD system has a high potential to save unwanted sample waste[7-9], it also requires a systematic dead volume to initiate experiments. Additionally, it involves the use of sophisticated machinery to generate micro-sized droplets and necessitates pilot experiments to determine the optimal parameters for data collection. The micro-sized nozzle of the DOD system is often prone to clogging due to impurities or sample aggregation, leading to the need for additional sample waste to replace the nozzle or reconfigure the system. It should be noted that the DOD system cannot be operated alone and requires an additional device to prevent the micro-sized droplets from dehydrating or experiencing severe temperature changes during data collection. These complex requirements for data collection present obstacles to efficiently obtaining the data and limit its potential

as a universal assay platform. Consequently, these approaches prove unsuitable for studying irreversible reactions due to their high sample consumption and practical challenges.

Design and material selection for the SXL fixed target system

Designing the SXL fixed target involved carefully considering how to handle liquid samples effectively. While our initial concept explored a microfluidic chip design known for its efficiency in capturing and positioning crystals[10], this approach was not suitable for SXL applications. In crystallography, the liquid acts as a carrier for the crystal sample of interest. In SXL, the liquid is the sample of interest, itself that needs to be preserved. Such a microfluidic design containing intra- and inter-connecting architectures requires a large liquid sample waste for the sample loading process. We then explored alternative fixed target designs that utilized discrete chambers for sample loading with no intra- and inter-connections[11-13]. These both-side opened chamber layouts may avoid the unwanted sample waste for the sample loading process, but they presented challenges in efficiently loading the sample and maintaining the intact environments of the liquid sample during data collection. Moreover, sealing the reaction chambers on both sides (one for loading and the other for removing the excess liquid buffer) would also be difficult, increasing the risk of leakage. This potential weakness in maintaining the intact environment of liquid samples poses severe challenges when rapid environmental changes occur during reaction progress. To adapt the fixed target for the liquid sample, we removed the connecting microfluidic components while simultaneously closing one side of the reaction chamber. This modification ensured stable sample preservation for data collection. To enhance the sample loading process, we could consider a square pyramidal shape of reaction chambers, which was developed for serial crystallography[13]. However, the edge structure near the center of the reaction chambers (pyramidal apexes) can significantly affect background scattering patterns, even with slight misalignment of the fixed target or fabrication imperfections. Therefore, we finally implemented truncated square pyramidal microchambers, having a flat surface on the bottom of the reaction chambers (100 μm × 100 μm), as shown in Fig. 1.

Unlike typical fixed targets for serial crystallography that often utilize various materials such as silicon nitride, PDMS, and other polymers, the SXL chip is fabricated from PDMS. This choice prioritizes maintaining a perfectly homogeneous and intact environment for the liquid sample during measurements. Obtaining a clean background scattering signal is crucial for accurate TR signal collection. PDMS fulfills these requirements for several reasons. PDMS's inherent adhesive nature facilitates a stable sealing environment when the thin film is pressed together, preventing leakage and maintaining sample integrity. Unlike other materials, PDMS allows for easy adjustment of its surface properties through various chemical treatments. By applying a surfactant to enhance the hydrophilicity of the PDMS and utilizing a truncated square pyramidal reaction chamber shape, we can facilitate the loading of aqueous samples. Furthermore, PDMS allows the SXL system to be applied in a wider range of solvent environments, extending its use beyond aqueous conditions. We envision its applicability to chemical reactions involving small molecules, where the liquid environments might be hydrophobic or aprotic. The easily controllable nature of PDMS hydrophobicity and hydrophilicity makes it ideal for such diverse applications. Moreover, PDMS offers a cost-effective solution with high precision and reproducibility. This is especially important for the SXL system, where repeated measurements are necessary to accumulate weak signals for difference scattering curve generation. PDMS ensures a stable and reliable measurement environment for consistent results. Studying reaction dynamics often involves using laser pumps, necessitating excellent optical clarity across a wide range of wavelengths, including UV light. PDMS provides outstanding transparency and stability across various wavelengths, allowing for clear observation of the reaction within the chip. PDMS exhibits high thermal stability, making it superior for tracking reaction dynamics across various temperature ranges. This is crucial for

experiments studying biological responses to temperature changes or observing chemical reactions at different temperatures. On the contrary, other materials, especially cyclic olefin polymers, cannot meet the previously described requirements for the application of SXL to liquid samples. Therefore, PDMS, with its advantageous properties, was chosen as the optimal material for the SXL chip, ensuring a reliable and stable measurement environment for diverse applications.

Ensuring flatness and reliable handling in the SXL chip design

To achieve the flatness of our SXL chip, we explored various materials for both the film and structure of the sample holder system, ultimately leading to the utilization of a polymer film (Chemplex Industries Inc., SpectroMembrane® 3024) with a carrier frame. This specialized film has been widely used as a sealing material and X-ray transparent observation window for the sample cup in X-ray fluorescence measurements, ensuring a flat surface and reliable sealing properties. The polymer film with a carrier frame, featuring a square paper frame along the edges, effectively prevents the formation of wrinkles on the thin film. Consequently, the use of this film significantly reduces the likelihood of bubbles or wrinkles appearing on the thin film SXL chip during sample loading. Furthermore, by employing a rigid paper frame to maintain the flatness of the SXL chip after sample loading, we ensure ease of handling. Additionally, the sample chip holder, detailed in Supplementary Fig. 3, incorporates a chip tray structure whose dimensions match the size of the chip and are carved out to the thickness of the SXL chip from the surface of the plate, to prevent unwanted movement or flexing of the SXL within the holder. Finally, upon assembly of the upper and lower plates of the sample chip holder, the excess film and paper frame are removed, and the tight coupling between the plates ensures that the film remains flat, as demonstrated in Supplementary Fig. 4. As shown in Fig. 2c, the static scattering profiles from each reaction chamber within the whole chip, show identical features across microchambers, confirming the uniform sample loading, integrity and flatness of the SXL chip.

As discussed in the main text, the hydrophilic nature of the SXL material (PDMS) and the wide-opening chamber design promote efficient sample loading. This design minimizes the risk of damage from capillary-induced physical contact during loading, as confirmed by the absence of observable damage from capillaries and the intact sample-loaded SXL chip after data collection (see Supplementary Fig. 4h). This feature ensures the chip's integrity and flatness, which are essential for obtaining reliable TR signals from the SXL chip.

The SXL method empowers an efficient and straightforward exploration of biological reactions.

Our use of consistent experimental setups, particularly the fixed target layout, for studying reaction dynamics across various demonstrative examples is noteworthy. Notably, only the laser pulse wavelength was varied, highlighting the flexible application of SXL across different targets. The SXL method serves not only as a universal assay framework for studying diverse biological reactions by minimizing unwanted sample waste but also provides advantages over conventional methods in data collection.

Conventional flow-cell systems encounter difficulties when investigating biological reactions with kinetics spanning beyond tens of milliseconds—a temporal range prevalent in various biological processes such as signaling cascades, DNA metabolism, and protein synthesis. In TR setups, the laser pulse is typically larger than that of the probe pulse to ensure accurate TR acquisition. In flow-cell systems, this configuration leads to prolonged waiting times between measurements to achieve complete sample replenishment, resulting in a reduction of the repetition rate. For instance, in a flow-cell system using laser and X-ray pulses with full-width at half-maximum (FWHM) sizes of 120 μm and 30 μm, respectively, to capture a time delay of 316 ms, the flow rate must be sufficiently low for

the region exposed to the laser pulse to be probed by the time-delayed X-ray pulse. Roughly estimating, the maximum flow rate can be calculated as ((120 − 30)/2) μm/316 ms = 140 μm/s. This calculation considers the difference between pulse lengths divided by two, assuming both pulses are aligned at the center position, ensuring a feasible tolerance for one-way sample flow is half of the difference. Subsequently, additional waiting time is required for the complete replenishment of the previously pumped sample portion, approximately three times the size of a laser pulse (given that the full size of the laser pulse is about three times the FWHM). Calculating this waiting time as (360 μm/2)/(140 μm/s) equals approximately 1.3 s. Consequently, the anticipated speed of data collection is expected to be less than 0.8 Hz. This trend is exacerbated when the time delay of interest extends beyond the mentioned timescale, resulting in a significant decrease in the efficiency of the data acquisition in flow-cell systems. In contrast, SXL collects time-delayed data of 316 ms at a rate of about 3 Hz (1/316 ms = 3.16 Hz, durations of the laser and X-ray pulses and stage movement can be negligible, less than a few milliseconds), because it does not require waiting for the sample replenishment of used portions but moves swiftly to the next individual microchamber. The disparity in repetition rates between the flow-cell and SXL methods becomes more pronounced when the time delay surpasses the mentioned timescale. Hence, SXL efficiently conducts data collection of reactions spanning moderate time scales, from milliseconds to seconds or minutes.

In a typical time-resolved measurement, data are collected at multiple time delays. It would be generally most efficient if a single time series containing all desired time delays suffices. If a long time delay (such as 316 ms explained in the previous paragraph) is included in the time series, it will result in slowing down the whole data collection due to the small repetition rate required to accommodate the long time delay. For this reason, to prevent inefficiencies in data collection with flow-cell systems, a common practice often involves segmenting the time ranges and conducting a series of data acquisitions for each segment of temporal ranges. This approach includes one for the fast time range covering femtoseconds to microseconds, another for milliseconds, and subsequent intervals. While this strategy appears to be effective in preventing undesired inefficiencies in data collection, its practical application demands supplementary, laborious pilot experiments for each segmented time series. In other words, to ensure acquiring reliable TR data, it is essential to precisely optimize the experimental parameters, such as flow speed, repetition rate of data collection and the length of the penetration of the laser and X-ray pulses, tailored to each segment within the specific time range. Additionally, it is inevitable to incorporate a common data point with the same time delay in each series to scale and merge the segments of collected data into an entire dataset. Dealing with this wide time range using flow-cell systems eventually requires a complex data collection scheme with a requirement of extra sample consumption. In contrast, SXL does not suffer from the need to lower the repetition rate for long time delays because it operates using the liquid sample contained in isolated, independent microchambers. Therefore, SXL allows efficient data collection without requiring such a strategy to cover a wide range of timescales, holding distinct advantages over flow-cell systems.

The SXL method enhances the temporal resolution of TR signals

The SXL method achieves a precise temporal resolution, highlighting the accurate TR signal compared to other sample delivery methods. In traditional TR setups for TRXL, such as those employing mixing devices, closed capillary and flow-cell systems, liquid samples are not contained in separate compartments. In this situation, the laser pulse cannot initiate the reaction across the entire sample content, posing a risk of diffusion from the pumped region (or the region where the reaction is initiated) into adjacent areas and vice versa, particularly when studying the milliseconds or second temporal range. In other words, the TR signal can be easily contaminated and altered due to the diffusion process. In contrast, the SXL approach stands out as the laser pulse easily triggers the reaction across the entire

sample content within a microchamber, completely isolated from neighboring microchambers. This superior layout guarantees homogeneity in the progress of the reaction throughout the entire sample, eliminating diffusion issues, and ultimately providing a TR signal not contaminated by diffusion with an accurate temporal resolution.

Future perspective of the SXL method using alternative X-ray sources

In this work, we demonstrated the application of the SXL platform at a time-resolved beamline specialized for time-resolved Laue crystallography (14IDB of APS). We anticipate that utilizing alternative X-ray beamlines and facilities such as microfocus X-ray beamlines and XFELs holds great promise for future SXL applications, particularly with regards to enhancement of sample utilization (for both microfocus X-ray and XFEL beamlines) and reliable data acquisition from diluted samples with a high SNR and temporal resolution (for XFEL beamlines). The successful acquisition of the DS signal from a small sample volume demonstrated in this study will be a significant advance in the field of TR techniques. This capability opens up new possibilities for studying complex biological reactions, particularly those that involve scarce or precious samples.

The potential for further miniaturization of the microchambers, enabled by the use of a micro-focused X-ray beam, holds promise for even more efficient and high throughput data collection. In this submitted work, we successfully fabricated a microchamber with a volume of four nanoliters, precisely tailored to match the dimensions of the currently achievable spatially overlapped X-ray and laser pulses. With the use of a micro-focused X-ray beam, it is possible to further reduce the microchambers by more than several tens of times, reaching the scale of a few hundred picoliters. The current design of our microchambers allows for sufficient spacing between them, enabling the fabrication of a positive master through a simple micro-milling process. There is great potential to decrease the spacing between the microchambers, accommodating a higher number of microchambers to be accommodated within the same dimensions of the SXL chip. This modification facilitates the collection of a comprehensive data set in a single batch using a single chip without employing multiple chips for additional measurements. For example, by fabricating a microchamber at half the size of the current layout (top: 0.05 mm, bottom: 0.11 mm and height 0.075 mm, and spacing: 0.23 mm, resulting in a microchamber volume of 0.5 nL) within the same chip dimensions, we can collect 4,800 TR data points using just 2.4 μL of sample. This demonstrates the potential to significantly increase the data acquisition capacity of our approach through the optimization of the microchambers and chip dimensions.

It is worth noting that a variety of biological reactions occur across a vast spectrum of timescales, ranging from microseconds to hours. For these reactions, highly specialized ultrashort X-ray pulses, which are less accessible to general users, are unnecessary. Instead, these reactions can be effectively investigated across a wider array of beamlines, including microfocus X-ray beamlines. As discussed in the main text, the SXL platform's adaptability extends beyond sample efficiency, and it can be readily adapted to various beamlines without requiring significant modifications to existing experimental setups in each beamline. This flexibility, combined with efficient sample utilization, positions SXL as a universal tool for studying a wide range of biological processes across diverse timescales at various beamlines. Moreover, even in scenarios where a pump-probe scheme is not available, slower reactions ranging from minutes to hours can be studied using SXL measurements with samples mixed with a reaction initiator or a photocaged molecule activated by a portable LED light source. Consequently, we anticipate that our SXL system can serve as a pivotal platform, enabling the study of diverse biological events for a broad spectrum of users through its efficiency enhancements and flexible capabilities.

To expand the applicability of the SXL to XFEL beamlines, it is crucial to maintain the structural integrity of the SXL during data collection, especially under the intense ultrashort pulses and high

photon flux characteristic of XFEL pulses. While the intense XFEL pulses raise concerns about damaging the SXL chip, its design offers inherent advantages. Even if a chamber is damaged by an XFEL pulse, the independent nature of the SXL chip's reaction chambers ensures unaffected subsequent measurements. We can simply move on to a fresh chamber within the chip using the single-probing scheme. In conclusion, the SXL chip's design and its compatibility with XFEL's "diffraction before destruction" principle[14] make it a promising candidate for future XFEL-based studies, offering superior temporal resolution and reduced sample consumption.

Supplementary References

1. Kim, J. G. et al. Mapping the emergence of molecular vibrations mediating bond formation, *Nature* **582**, 520–524 (2020).
2. Levantino, M., Yorke, B. A., Monteiro, D. C., Cammarata, M. & Pearson, A. R. Using synchrotrons and XFELs for time-resolved X-ray crystallography and solution scattering experiments on biomolecules. *Curr. Opin. Struct. Biol.* **35**, 41–48 (2015).
3. Ki, H., Oang, K. Y., Kim, J. & Ihee, H. Ultrafast X-ray crystallography and liquidography. *Annu. Rev. Phys. Chem.* **68**, 473–497 (2017).
4. Yamada, Y., Matsuo, T., Iwamoto, H. & Yagi, N. A compact intermediate state of calmodulin in the process of target binding. *Biochemistry* **51**, 3963–3970 (2012).
5. Josts, I. et al. Photocage-initiated time-resolved solution X-ray scattering investigation of protein dimerization. *IUCrJ* **5**, 667–672 (2018).
6. Arnlund, D. et al. Visualizing a protein quake with time-resolved X-ray scattering at a free-electron laser. *Nat. Methods* **9**, 923–926 (2014).
7. Graceffa, R., Accardo, A., Rosenthal, M., Vlasova, M. & Riekel, C. Perspectives: DOD inkjets at high and ultra-brilliant light sources. *NanoWorld J.* **4**, 54–60 (2019).
8. Guo, Q. et al. A review on acoustic droplet ejection technology and system. *Soft Matter* **17**, 3010–3021 (2021).
9. Zheng, Y. et al. Miniaturizing chemistry and biology using droplets in open systems. *Nat. Rev. Chem.* **7**, 439–455 (2023).
10. Rizzini, L. et al. Perception of UV-B by the Arabidopsis UVR8 protein. *Science* **332**, 103–106 (1990).
11. Kliebenstein, D. J., Lim, J. E., Landry, L. G. & Last, R. L. Arabidopsis UVR8 regulates ultraviolet-B signal transduction and tolerance and contains sequence similarity to human regulator of chromatin condensation 1. *Plant Physiol.* **130,** 234–243 (2002).
12. Christie, J. M. et al. Plant UVR8 photoreceptor senses UV-B by tryptophan-mediated disruption of cross-dimer salt bridges. *Science* **335**, 1492–1496 (2012).
13. Wu, D. et al. Structural basis of ultraviolet-B perception by UVR8. *Nature* **484**, 214–219 (2012).
14. Chapman, H. et al. Femtosecond X-ray protein nanocrystallography. *Nature* **470**, 73–77 (2011).
